# Editorial Note: Predicting corporate management performance using AI: Incorporating CEO strategy insights from sustainable management reports

**DOI:** 10.1371/journal.pone.0357856

**Published:** 2026-09-09

**Authors:** The PLOS One Editors

The *PLOS One* Editors issue this Editorial Note to inform readers that work cited in this article [1] as Reference 27 was retracted before the publication of [1], and that the work cited as Reference 55 was retracted after the publication of [1]. PLOS considers that these references are not crucial in supporting the research or conclusions reported in [1].

Additionally, the authors provided the following statement regarding their use of AI tools and technologies for the preparation of [1]:

OpenAI's ChatGPT was used solely for language-related assistance during manuscript preparation, specifically for English translation and language editing of text originally drafted by the authors.

The authors carefully reviewed and revised all AI-assisted language outputs to ensure that they accurately reflected the intended meaning of the original text and the scientific content of the manuscript. All final decisions regarding the wording and content of the manuscript were made by the authors.

ChatGPT was not used to generate or modify the study data, perform statistical analyses, develop the research methodology, generate or interpret the study results, or create supporting data or analytical outputs. Its use was limited to English translation and language editing of the manuscript text.

The authors take full responsibility for the accuracy and integrity of the final published content.
